# Hansen Disease among Micronesian and Marshallese Persons Living in the United States

**DOI:** 10.3201/eid1707.102036

**Published:** 2011-07

**Authors:** Patricia Woodall, David Scollard, Latha Rajan

**Affiliations:** Author affiliations: Tulane University, New Orleans, Lousiana, USA (P. Woodall, L. Rajan);; National Hansen’s Disease Program, Baton Rouge, Louisiana, USA (D. Scollard)

## Abstract

An increasing proportion of Hansen disease cases in the United States occurs among migrants from the Micronesian region, where leprosy prevalence is high. We abstracted surveillance and clinical records of the National Hansen’s Disease Program to determine geographic, demographic, and clinical patterns. Since 2004, 13% of US cases have occurred in this migrant population. Although Hawaii reported the most cases, reports have increased in the central and southern states. Multibacillary disease in men predominates on the US mainland. Of 49 patients for whom clinical data were available, 37 (75%) had leprosy reaction, neuropathy, or other complications; 17 (37%) of 46 completed treatment. Comparison of data from the US mainland with Hawaii and country-of-origin suggests under-detection of cases in pediatric and female patients and with paucibacillary disease in the United States. Increased case finding and management, and avoidance of leprosy-labeled stigma, is needed for this population.

At 11 cases per 10,000 population and 8 per 10,000, respectively, in 2007, the small Pacific Island nations of the Republic of the Marshall Islands and the Federated States of Micronesia have the highest prevalence of Hansen disease (HD), i.e., leprosy, in the world and have made little progress in the past decade toward the World Health Organization (WHO) leprosy elimination target of <1 per 10,000 (*1**,**2*). During the first quarter of 2010, 33 new cases were detected among the 54,000 residents of the Marshall Islands (*3*).

HD has been present in the United States for more than a century; the number of patients has remained relatively constant at 150–200 per year (*4*).The US National Hansen’s Disease Program (NHDP) has noted an increasing number of cases among US-resident Marshall Islanders and Micronesians, including several persons with advanced disease. In 1996, the Hawaii HD program reported a cluster involving 16 (5%) of 321 persons screened from a Marshallese migrant community (*5**,**6*). In 2002, the US Army noted 3 cases in 1 month in soldiers from this region (*7*). The recent reporting of multiple cases among the Marshallese community in northwestern Arkansas (Centers for Disease Control and Prevention, unpub. data, 2006) has drawn attention in a region unaccustomed to leprosy, with its stigmatizing historical connotations (*8**,**9*).

Under the terms of the Compacts of Free Association (the legal documents governing the relationships between the United States, Federated States of Micronesia, and the Republic of the Marshall Islands), citizens of this former US Trust Territory of the Pacific Islands are not subject to usual immigration requirements but may freely enter, reside, and work in the United States for as long as they wish. They hold a unique legal status, are not classified as immigrants, and maintain their country-of-origin citizenship. Transportation data indicate net emigration of an average of 952 Marshallese and 1,443 Micronesians annually, with a total of 38,325 emigrants for 1991–2006; almost all of these persons are thought to have immigrated to the United States and its territories (*10*). The actual distribution of this population within the United States is unknown; a specific category included in the 2010 US Census should provide this information. As economic and climatologic pressure drive increasing emigration from this HD-endemic former US Trust Territory, the US HD case load is expected to continue to increase, worsening health disparities and requiring increased program and local resources, although this increased case load is unlikely to create a public health threat of transmission to the general population. Cultural and socioeconomic issues may affect case detection and long-term disease management in this population, including adherence to and completion of therapy.

The objective of this report is to describe, on the basis of secondary analysis of existing program data, the epidemiology of HD among Marshallese and Micronesian persons residing in the United States. The intent is to assist in providing resources to address a health disparity that disproportionately affects a group of a particular national origin, while at the same time avoiding worsening of ethnic and disease-related stigma.

## Materials and Methods

Demographic and disease-related data were abstracted for January 1990–October 2009 NHDP surveillance and clinical records of cases that identified patients’ place of birth as the former Trust Territory (Marshall Islands or Micronesia). To facilitate global comparison, US cases (reported by using the Ridley-Jopling classification system [11]) were reclassified into the WHO multibacillary/paucibacillary system (*12*). Country-of-origin HD data (case numbers or rates and age/sex/disease-classification) were abstracted from WHO reports (*1**,**2**,**13*). Country-of-origin demographic data, US Census reports, and published reports relevant to migration patterns were abstracted to obtain denominator estimates where possible (*14**–**17*). Data were analyzed by using the SPSS version 16.0 statistical analysis program (SPSS Inc., Chicago, IL, USA). The study was approved by the Tulane University Institutional Review Board and exempted from review by the University of Arkansas for Health Sciences Institutional Review Board.

## Results

**Figure 1:**
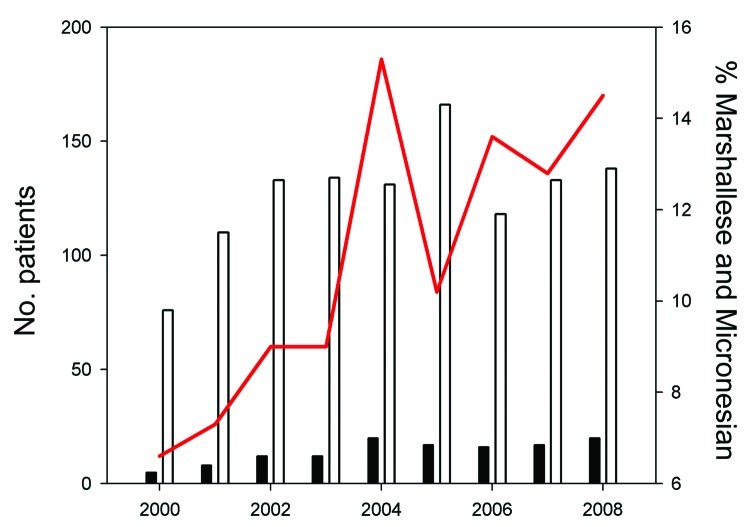
New patients of Marshallese or Micronesian origin with Hansen disease, compared with total new US patients with Hansen disease, 2000–2008. White bars, total US patients; black bars, total patients of Marshallese or Micronesian origin; red line, patients of Marshallese or Micronesian origin as percentage of total US patients.

The number of HD case-patients of Micronesian or Marshallese origin as a proportion of all US cases has risen over the past decade, with Micronesian and Marshallese patients constituting 90 (13%) of all 686 cases in the United States during 2004–2008 (Figure 1); the total source population of these 2 countries is only ≈170,000. Data are summarized in Table 1.

**Table 1:** Hansen disease in residents of Micronesian and Marshallese origin, United States*

Population group	No. (%) patients				Median time from US entry to report, y (range)	MDT,† no. (%)	Complications, no. cases					
	Total	MB	F	Age <15 y			ENL	RR	Hand/foot neuropathy	Eye	Other	Any (%)‡
Hawaii-residing Micronesians, 2000–2009§	29	22 (76)	5 (17)	3 (10)	1.5 (0–13)	NA	NA	NA	NA	NA	NA	NA
Hawaii-residing Marshallese, 2000–2009§	43	31 (72)	17 (39)	1 (2)	1 (0–14)	NA	NA	NA	NA	NA	NA	NA
US Mainland Micronesians, 1990–2009	55	42 (76)	10 (18)	6 (10)	3 (0–10)	10 (42)	15	3	8	6	3¶	25 (77)
US Mainland Marshallese, 1990–2009	33	25 (76)	11 (33)	3 (10)	2 (0–12)	7 (32)	6	3	5	1	1#	12 (75)
Arkansas-residing Marshallese, 1990–2009	17	15 (88)	5 (30)	0 (0)	2 (0–5)	3 (20)	4	2	2	0	0	12 (70)

*Data from National Hansen’s Disease Program surveillance and clinical records, January 1990–October 2009. MB, multibacillary; MDT, multidrug therapy; ENL, erythema nodosum leprosum; RR, reversal reaction; NA, data not available. †MDT completed, excluding patients currently under active treatment. ‡Patients for whom clinical data were available. §Pre-2000 data were not reported to National Hansen’s Disease Program. ¶1 renal, 1 testicular, 1 palatal. #1 renal.

During January 2000–October 2009, 72 (55%) of US HD cases in Micronesian or Marshallese patients were reported in Hawaii. Of 29 Micronesian patients, 22 (76%) had multibacillary HD, 5 were female, and 3 were children. Of 43 Marshallese patients, 31 (72%) had multibacillary HD, 17 (17%) were female, and 1 was a child. Cases in Hawaii were not consistently reported to NHDP until the late 1990s; however, Bomgaars et al. reported that HD cases in 15 Micronesian and 23 Marshallese patients were noted there from 1990 through mid-1998, including the Marshallese cluster summarized in Table 2 (*6*).

**Table 2:** Hansen disease patient data for multibacillary disease status, female sex, and age <15 y, as reported by sources other than the US National Hansen’s Disease Program*

Data source	Multibacillary disease, %	Female sex, %	Age <15 y
Federated States of Micronesia national data, 2000–2007†	44	35	34
Federated States of Micronesia Leprosy Elimination Campaign, 2005‡	30	36	32
Republic of the Marshall Islands national data, 2000–2007†	55	42	23
Republic of the Marshall Islands Leprosy Elimination Campaign, 1999‡	31	NA	35
Hawaii Marshallese cluster, 1998‡	28	NA	39

*NA, data not available. †World Health Organization annual reports (national surveillance). ‡Published reports from active case-finding campaigns (*5**,**13**,**18**–**20*).

**Figure 2:**
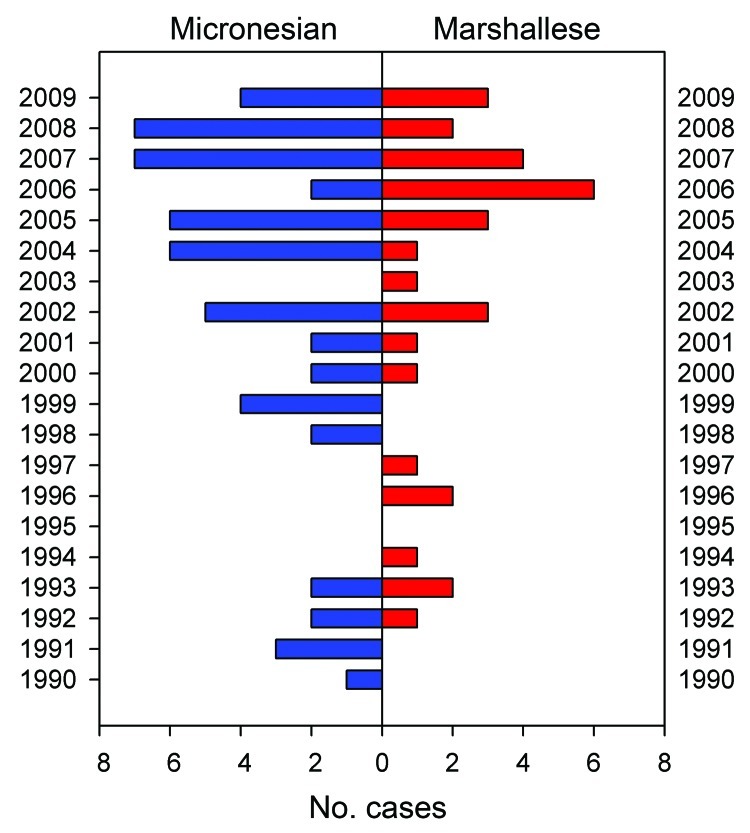
Hansen disease in patients of Marshallese or Micronesian origin, US mainland, by year reported, January 1990–October 2009.

**Figure 3:**
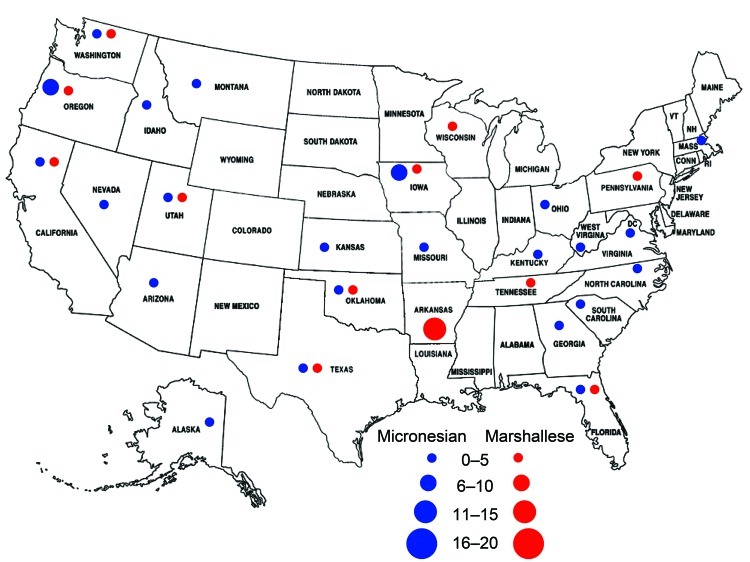
States reporting Hansen disease in patients of Marshallese or Micronesian origin, US mainland, January 1990–October 2009. The number of cases reported from each state is indicated.

Since 1990, 88 cases have been reported in 26 states of the US mainland (Figures 2, 3), 66 of these since 2000. Cases are increasingly being reported in inland locations. In all groups, the majority of patients were young men with multibacillary disease.

Among Micronesians, 55 cases occurred in 22 states of the US mainland (Table 1), 41 of these since 2000. All but 2 patients were <40 years of age, 42 (76%) had multibacillary disease, 10 (18%) were female, and 6 (10%) were children. Six patients reported having a previous HD diagnosis in the Federated States of Micronesia; 4 of these patients reported having completed the WHO-recommended 1 year of multidrug therapy (MDT) there. Of these patients, 14 (25%) originated in Chuuk State and 13 (24%) in Pohnpei State; for 28 (51%), the specific Federated State was not documented. During the 1990s, 11 of 14 US-mainland HD cases were reported in the western coastal states of California, Washington, and Oregon. Since 2000, a total of 28 of 41 cases were reported in midwestern and southern states (e.g., 6 in Iowa, 5 each in Texas and Florida, 3 in Missouri). Before moving to the reporting state, 21 patients had resided in other mainland states (often in multiple locations), 5 in Hawaii, and 8 in Guam. Of the 33 patients for whom clinical data were available, 25 (76%) had >1 complication, including 15 patients with erythema nodosum leprosum (ENL) and 3 with severe facial involvement (lagophthalmos, blindness, nasopalatal destruction). One patient was pregnant when her HD was diagnosed. Of the 24 patients for whom pharmacy data were available (excluding 8 who had recently begun treatment), the complete US-recommended MDT course had been dispensed to 10 (42%) (Table 1).

Of the 33 US mainland Marshallese patients since 1990 (25 of these since 2000), single cases were reported in 9 states, 2 in California, 5 in Washington, and 17 in Arkansas. All but 1 patient were <40 years of age, 25 (76%) had multibacillary disease, 11 (33%) were female, and 3 (10%) were children. Three patients reported that their HD had been diagnosed in the Marshall Islands; none had completed MDT there. Nine patients had resided in other mainland states before moving to the reporting state; 5 had first lived in Hawaii. Of the 16 patients for whom clinical data were available, 12 had >1 complication, including 6 patients with ENL. Two patients were pregnant at the time of diagnosis. Of the 22 patients for whom pharmacy data were available (excluding 3 currently being treated), the complete US-recommended MDT course had been dispensed to 7 (32%) (Table 1).

Beginning in 1996, 17 HD cases have been reported among the ≈8,000–10,000 Marshallese living in northwestern Arkansas, 10 of these since 2004. All patients were <40 years of age, 15 (88%) had multibacillary disease, 5 (30%) were female, and none were children. (Two 25-year-old men with multibacillary disease, reported in November 2009 after completion of data collection for this study, are not included in this analysis.) Four patients had previously lived in other states, none in Hawaii. Specific island origin within the Marshall Islands was not reported; however, the general Arkansas Marshallese population is known to come from several different atolls. Of the 10 patients for whom clinical data were available, 7 had >1 complication, including 4 patients with ENL. Excluding the 2 patients currently being treated, the US-recommended MDT course had been dispensed to 3 (20%) (Table 1).

**Figure 4:**
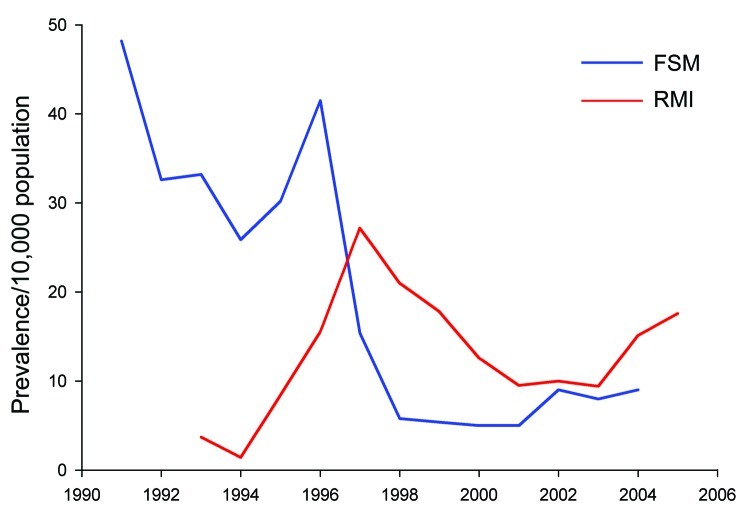
Hansen disease prevalence as cases per 10,000 population, Federated States of Micronesia (FSM) and Republic of the Marshall Islands (RMI) 1991–2005 (*1**,**2**,**13*).

**Figure 5:**
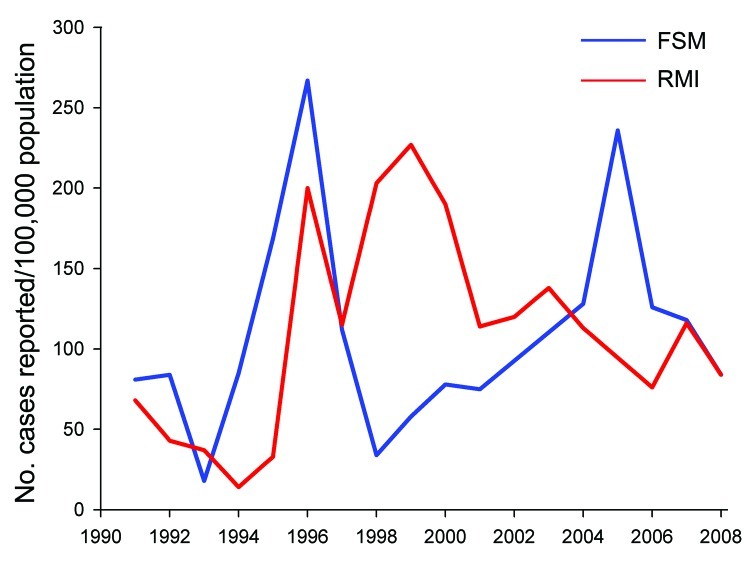
Hansen disease cases per 100,000 population, Federated States of Micronesia (FSM) and Republic of the Marshall Islands (RMI) (*1**,**2**,**13*), 1990–2008.

HD prevalence reported in the countries of origin is summarized in Figures 4 and Figure 5. Because of the epidemiologic characteristics of HD (e.g., lack of biomarker, prolonged latency), surveillance is generally based on program records rather than population surveys; prevalence and case-detection rate (an approximation of incidence) are highly dependent on operational factors (Figure 5). Active case-detection programs were done in the Federated States of Micronesia in 1996 and 2005 and in the Marshall Islands in 1996 and 1998–1999 (*18**–**20*). Patterns of age/sex/classification distribution differed markedly during these years, with more children and paucibacillary disease being identified than during the usual passive surveillance years (Table 2); this pattern was also noted in the 1998 Marshallese cluster in Hawaii.

## Discussion

This study was descriptive; thus, comparisons among the various population subgroups are not statistically valid because of small numbers, varied data sources, and unknown age/sex distribution and denominators for the overall migrant populations. However, because age and sex distributions for the Arkansas, Hawaii, and Republic of the Marshall Islands Marshallese populations are similar (*17*), some crude comparisons can be made for these groups. Unless an unidentified confounder is present, rates and distribution of cases would be expected to be similar in the 3 groups.

**Figure 6:**
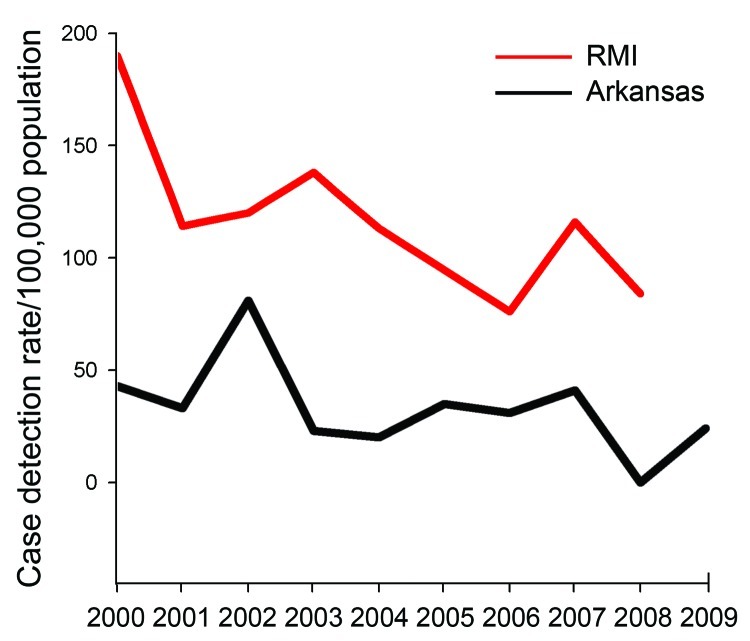
Estimated Hansen disease cases per 100,000 population, Arkansas Marshallese and Republic of the Marshall Islands (RMI), 2000–2009. RMI rates from World Health Organization reports (*1**,**2**,**13*); Arkansas cases from National Hansen’s Disease Program records. Arkansas Marshallese population (denominator) derived from US Census estimates (*15**,**17*).

The Arkansas and Hawaii Marshallese populations were approximately the same size (3,000 each) and age/sex distribution (median age 20 years, M:F = 1) in 1998–2001; rough estimates place the current Arkansas population at 8,000–10,000 and Hawaii at 6,000–8,000. However, Hawaii has identified 2.6× more cases since 2000, more female patients, and more paucibacillary disease and has found disease in children. Estimated case detection rates for Arkansas Marshallese persons are approximately half those for the Marshall Islands (Figure 6) and more skewed toward multibacillary disease, male, and adult patients (Table 1). This Arkansas and general US mainland pattern of predominantly multibacillary disease in men does not reflect disease distribution patterns in the countries of origin or in Hawaii, suggesting under-detection of child, female, and paucibacillary disease patients rather than a biologic disease pattern (Tables 1, 2).

Of 49 mainland patients from both the Federated States of Micronesia and the Marshall Islands for whom clinical data were available, 37 (75%) had >1 complication, such as leprosy reaction, neuropathy, or other tissue involvement (e.g., palatal, renal, testicular). Although this finding could certainly reflect reporting bias, with the most severely affected being more likely to seek care and to be reported, it does indicate the magnitude of the need for treatment resources for this population with limited health care access. The most severe cases of blindness and disfigurement occurred in Micronesians living alone or in very small ethnic communities, limiting the potential impact of ethnic-targeted services. The high number of ENL cases is of note because these patients may require corticosteroid treatment for many years. The low rates of MDT completion may contribute to poor clinical outcomes and possibly to drug resistance and ongoing transmission.

This study has several limitations. Hawaii did not report to the NHDP system until the late 1990s. Cases in Guam and Northern Marianas and among US military personnel are not reported to the NHDP. Reporting is not mandatory in all states but is likely to be fairly complete because of US physicians’ need for consultation on management of this unfamiliar condition. Since the 2004 US Food and Drug Administration designation of clofazimine as an investigational drug, any patients with multibacillary disease are likely to have been reported so they would be able to receive the recommended MDT, although paucibacillary disease may be underreported. The increase in case reports since 2004 could reflect this requirement.

Measures of adherence and completion of treatment, complications, and other clinical data were available only for mainland patients receiving direct or pharmacy care from the Baton Rouge NHDP facility. MDT is dispensed directly to active patients or provided to local physicians or health departments for supervision of treatment. Although adherence to and completion of treatment are monitored at national program level for HD patients attending clinics funded through contracts with the NHDP, for whom compliance exceeds 90% (D. Scollard, unpub. data), this level of follow-up has not been possible with private physicians, who are the providers for all of the Marshallese and Micronesian patients. In this situation, dispensing of MDT by the NHDP pharmacy was the only treatment measure available; actual adherence to treatment is unknown but is likely to be much lower and could be a subject for future study. A variety of cultural factors are likely to contribute to the low rate of treatment completion in this group, but evaluation of these cultural issues is beyond the scope of this study

Since achievement of the global leprosy elimination target of <1 case per 10,000 persons, attention has waned in some regions in which this neglected tropical disease persists. Although prevalence in the Republic of the Marshall Islands and the Federated States of Micronesia fell during the 1990s, rates have remained stable at high levels for the past decade. In these nonindustrialized nations, leprosy receives low priority in relation to more urgent public health concerns (rising rates of multidrug-resistant tuberculosis, 30% adult prevalence of type 2 diabetes, high infant mortality, and many others). Under the Compacts of Free Association, since the 1986 independence of the Federated States of Micronesia and the Republic of the Marshall Islands most health funding to these former territories flows from the United States. Assistance with leprosy control in the countries of origin would, in addition to improving health conditions there, be the first step toward preventing importation of cases to the United States. Because of the unique status of migrants from these nations to the United States, Micronesian and Marshallese migrants receive neither the health screening required for immigrants (which is not effective at preventing importation of HD because of its decades-long incubation period) nor targeted health services often available to large immigrant or refugee populations. Although legally residing in the United States, they remain citizens of their home nations and are thus ineligible for US health programs such as Medicare and Medicaid, although NHDP services such as biopsy interpretation, MDT, and direct outpatient care at the Baton Rouge facility are available. Many remain uninsured (>50% of the Arkansas Marshallese population) (*17*). Mainland state and local health departments are not typically resourced to serve either this population or this otherwise-rare condition. Hawaii receives more than $15 million annually in Compact impact funds to partially address the multiple health issues of the Marshallese and Micronesian migrant populations, but these additional US funds have not been available to the mainland states (*21*).

With the goal of decreasing health disparity and preventing disability, case-finding and case-management interventions are needed in US-resident Marshallese and Micronesian communities that are integrated into general health services and avoid the stigma of leprosy-labeled activities. Special efforts may be necessary to increase case detection among women and children. Large populations, such as the Arkansas Marshallese community, may be easier to reach with targeted but nonstigmatizing efforts, as has occurred in Hawaii. The small, widely distributed Micronesian communities, where the most severe, disabling cases have been identified, may be more difficult to reach.
