# Errata—Vol. 17, No. 7

**DOI:** 10.3201/eid1709.C41709

**Published:** 2011-09

In the article Hansen Disease among Micronesian and Marshallese Persons Living in the United States (P. Woodall et al.), the first paragraph of the Results section should reference 686 total cases of Hansen disease in the United States. The article has been corrected online (www.cdc.gov/eid/content/17/7/1202.htm).

